# Molecular diversity of *Mycobacterium tuberculosis *isolates from patients with tuberculosis in Honduras

**DOI:** 10.1186/1471-2180-10-208

**Published:** 2010-08-03

**Authors:** Senia Rosales, Lelany Pineda-García, Solomon Ghebremichael, Nalin Rastogi, Sven E Hoffner

**Affiliations:** 1 Escuela de Microbiología, Universidad Nacional Autónoma de Honduras, Tegucigalpa, Honduras; 2 Division of Global Health (IHCAR), Department of Public Health Sciences, Karolinska Institutet, Stockholm, Sweden; 3 Department of Bacteriology, Swedish Institute for Infectious Disease Control, Solna, Sweden; 4 Department of Microbiology, Tumor and Cell Biology (MTC), Karolinska Institutet, Stockholm, Sweden; 5 Unité de la Tuberculose et des Mycobactéries, Institut Pasteur de la Guadeloupe, Abymes, Guadeloupe

## Abstract

**Background:**

Tuberculosis persists as a public health problem in Honduras. A better knowledge of the molecular characteristics of *Mycobacterium tuberculosis *strains will contribute to understand the transmission dynamics of the disease within the country. The aim of this study was to provide an insight of the genetic biodiversity of *M. tuberculosis *clinical isolates collected in Honduras between 1994 and 2002. Genotyping was performed using spoligotyping and RFLP. The spoligotypes obtained were compared with the SITVIT2 proprietary database of the Pasteur Institute of Guadeloupe.

**Results:**

Spoligotyping grouped 84% of the isolates into 27 clusters (2 to 43 strains per cluster). Of the 44 shared international types (SITs) identified among the Honduran stains, 8 SITs were newly identified either within the present study or after match with an orphan type previously identified in the SITVIT2 database. In addition, 16 patterns corresponded to orphan, previously unreported isolates.

The Latin American Mediterranean (LAM) lineage was the most common in this study; 55% of the strains belonged to this family. Other genotypes found were Haarlem (16%), T (16%), X-clade (6%), Unknown signature (5%) and S (1%). Only one Beijing strain was identified (0.5%).

We observed a high degree of diversity after characterizing the 43 isolates belonging to the main spoligotyping cluster (SIT 33, LAM3) with IS*6110*-RFLP. A total of 35 different RFLP-fingerprints were detected, of which 6 patterns corresponded to the same number of clusters comprising 14 strains.

**Conclusions:**

The findings obtained in this study show that tuberculosis transmission in Honduras is due to modern *M. tuberculosis *lineages with high level of biodiversity.

## Background

Honduras is the heart of Central America. It has a population of 8 million inhabitants [1] and is located between the Caribbean Sea and the Pacific Ocean sharing boundaries with Guatemala, El Salvador and Nicaragua. As in many other low-income countries, tuberculosis (TB) is a major public health issue. Although the reported TB incidence rate has decreased from 72/100,000 in 1993 to 37/100,000 in 2008 [2], TB control remains a priority. A better understanding of TB transmission in the country could help to identify risk settings as well as to improve contact tracing.

Since the early 1990's new DNA-fingerprinting tools have been developed to improve TB case detection and control [3-5]. Molecular typing techniques have been used to detect and follow the spread of individual strains of the *Mycobacterium tuberculosis *complex (MTC), complementing conventional epidemiological methods and allowing the study of transmission dynamics. Among these techniques is the restriction fragment length polymorphism (RFLP), it uses the insertion sequence IS*6110 *as a probe to enable strain differentiation, and has been considered the gold standard for genotyping the MTC [6].

Another molecular fingerprinting method is spoligotyping, a robust polymerase chain reaction (PCR) - based technique which relies on the detection of 43 short non-repetitive spacer sequences located in the Direct Repeat (DR) region of the MTC genome [7].

A first overview of the population structure of MTC strains circulating in Honduras was reported in a study conducted in 1996 [8]. In this study, a high degree of strain diversity, based on RFLP molecular fingerprinting was seen among 84 *M. tuberculosis *isolates obtained from the same number of Honduran pulmonary-TB patients.

The purpose of this study was to provide a better insight of the biodiversity of Honduran MTC isolates using the spoligotyping as the genotyping technique.

## Methods

### Study population

The study population consisted of 206 clinical *Mycobacterium tuberculosis *isolates from Honduran TB patients. These were collected at two different time points. Eighty-seven strains (group I) were isolated between 1994 and 1998 at the Instituto Nacional Cardiopulmonar (INCP), the national reference hospital for lung and heart diseases. Part of this material was also included in the first molecular typing study carried out in Honduras [8]. The remaining 119 strains (group II) were collected countrywide in 2002 as part of the National Survey of Tuberculosis Drug-Resistance [9], coordinated by the Honduran National TB Reference Laboratory (NRL).

All strains were isolated on Lowenstein Jensen (LJ) medium and confirmed to be of the MTC using standard biochemical tests [10] (niacin production, catalase activity and nitrate reduction). The drug-susceptibility profile of the isolates belonging to group I was determined at the Swedish Institute for Infectious Disease Control (SMI) using the BACTEC 460 system (Becton Dickinson, Sparks, MD USA) [11], with the following drug concentrations: rifampicin (RIF) 2.0 μg/ml, isoniazid (INH) 0.2 μg/ml, streptomycin (STM) 4.0 μg/ml and ethambutol (EMB) 5.0 μg/ml. For the group II isolates, the proportion method on LJ medium [12] was performed at the Honduran NRL to determine the susceptibility to the first-line drugs. The following critical concentrations were used: RIF 40 μg/ml, INH 0.2 μg/ml, STM 4.0 μg/ml, EMB 2.0 μg/ml.

The strains were subsequently sent to the SMI, where the genotyping was performed.

### DNA extraction

All isolates were subculture on LJ medium at SMI. For spoligotyping, mycobacterial lysates were prepared by resuspending 2 loops of bacteria in 250 μl of 1 × TE buffer. After heat-killing the cells at 80°C during 1 hour, the suspensions were centrifuged at 13000 rpm for 2 minutes. Supernatants were discarded and pellets resuspended in 500 μl of 150 mM NaCl, These centrifugation and resuspension steps were repeated. The final pellet was then dissolved in 25 μl of 1 × TE buffer.

For RFLP typing, genomic DNA was obtained using the cetyl-trimethyl ammonium bromide (CTAB) method [13].

### Spoligotyping

All isolates were genotyped with a spoligotyping commercial kit (Isogen Bioscience, BV Maarsen, The Netherlands) according to the protocol previously described by Kamerbeek *et al *[7]. Briefly, the DR region of the TB genome was amplified using primers DRa and DRb, and the amplified biotinylated products hybridized to a set of 43 oligonucleotides covalently bound to a membrane. The hybridized PCR products were then incubated with a streptavidin-peroxidase conjugate and the membrane then exposed to chemiluminescence (Amersham ECL Direct™ nucleic acid labeling and detection system, GE Healthcare Limited, UK) and exposed on an X-ray film (Amersham Hyperfilm™ ECL, GE Healthcare Limited, UK) according to the manufacturer's instruction. The X-ray film was developed using standard photochemical procedures after 20 minutes exposure. DNA extracts of *M. tuberculosis *H37Rv and *M. bovis *BCG were used as controls.

The patterns obtained were analyzed using the BioNumerics software version 5.1 (Applied Maths, Sint-Martens-Latem, Belgium). A cluster was defined as two or more strains sharing identical spoligotyping patterns. Spoligotypes in binary format were converted to an octal code for comparison with the SITVIT2 proprietary database of the Pasteur Institute of Guadeloupe, which is an updated version of the previously released SpolDB4 database [14,15].

### Database comparison and geographical distribution of spoligotypes

The obtained octal spoligotypes codes were entered into the SITVIT2 database. In this database, two or more patient isolates sharing identical spoligotype patterns are define as SIT (Spoligotype International Type) whilst single spoligopatterns are defined as "orphan" isolates. Major phylogenetic clades were assigned according to signatures provided in SpolDB4. The SpolDB4 defines 62 genetic lineages/sub-lineages [14] and includes specific signatures for various *M. tuberculosis *complex members such as *M. bovis, M. caprae, M. microti, M. canettii, M. pinipedii*, and *M. africanum*, as well as including rules for defining the major lineages/sub-lineages for *M. tuberculosis sensu stricto*. At the time of the present study, SITVIT2 contained more than 3000 SITs with global genotyping information on around 74,000 *M. tuberculosis *clinical isolates from 160 countries of origin.

The worldwide distribution of predominant spoligotypes found in this study (SITs representing 4 or more strains) was further investigated using the SITVIT2 database, and regions with ≥5% of a given SIT as compared to their total number in the SITVIT2 database, were recorded. The various macro-geographical regions and sub-regions were defined according to the specifications of the United Nations [16].

The same criteria were used to compare the distribution by country of predominant SITs (countries with ≥5% of a given SIT). The three-letters country codes were used as defined in the ISO 3166 standard [17].

### Comparison of spoligotypes families and principal genetic groups

The overall distribution of strains, according to the major *M. tuberculosis *spoligotyping-defined families, was compared to the principal genetic groups (PGG) based on *KatG463-gyrA95 *polymorphisms [18]. The comparison was inferred from the reported linking of specific spoligotype patterns to PGG1, 2 or 3 [19-21].

### Restriction fragment length polymorphism

The standard RFLP protocol [6] was used to further characterize 43 strains found to belong to a single spoligotype cluster. Briefly, the genomic mycobacterial DNA was digested by the restriction enzyme *Pvu *II and separated by gel electrophoresis. Following southern blot, samples were hybridized with the probe IS*6110 *and detected by chemiluminescence (Amersham ECL direct™ nucleic acid labeling and detection system, GE Healthcare Limited, UK) using X-ray films (Amersham Hyperfilm™ ECL, GE Healthcare Limited, UK).

The *M. tuberculosis *strain 14323 was used as an external marker for the comparison of patterns and the BioNumerics software was used to analyze the patterns obtained. A dendrogram was constructed to show the degree of similarity among the strains using the un-weighted pair group method of arithmetic average (UPGMA) and the Jaccard index (1% tolerance, 0.5% optimization). Two or more strains with exactly the same fingerprint were defined as an RFLP cluster.

### Epidemiological information

General epidemiological data such as sex, age, geographic origin, HIV status, previous history of TB and drug-susceptibility profile was retrieved from laboratory records and/or medical files, using a standardized questionnaire.

### Statistical analysis

Epi Info™ version 3.5.1 (Centers for Disease Control, Atlanta, USA) was used for the statistical analysis of the data. The association between demographic characteristics and clustering by spoligotyping was assessed using Yates-corrected Chi square (X^2^) or Fisher exact (2-tailed) tests; p- values < 0.05 were considered significant. Odds ratios (OR) and the 95% confidence interval (95% CI) were calculated. Statistical analysis of mean ages was performed using the Student's t- test.

### Ethical considerations

This study received approval from the Ethical Committee in Biomedical Research of the Scientific Research Unit, of the Universidad Nacional Autónoma de Honduras.

## Results

### Spoligotyping results

The 206 *M. tuberculosis *isolates from this study belonged to one of 60 different spoligotype patterns. Sixteen patterns corresponded to orphan strains that were unique among more than 74,000 strains recorded in the SITVIT2 database (Additional file 1) whilst 44 patterns, from 190 patient isolates, corresponded to shared-types, i.e. they had an identical pattern shared with two or more patient isolates worldwide (within this study, or matching another strain in the SITVIT2 database). An SIT number was attributed to each pattern according to the SITVIT2 database. As shown in additional file 2, among the 44 identified SITs, a total of 36 SITs (containing 173 isolates) matched a pre-existing shared type in the SITVIT2 database, whereas 8 SITs (containing 17 isolates) were new, either within the present study or after a match with an orphan in the database.

Among the 60 spoligotypes patterns characterized in the present study, 27 patterns corresponding to clusters with 2-43 isolates per cluster were identified, accounting for a very high clustering rate of 84% (173/206).

Linking the spoligotyping results and clade definitions to the distribution of clinical isolates within PGG1 versus PGG2/3 (the latter being easily characterized by the lack of spacers 33-36), showed that TB in Honduras is exclusively caused by modern tubercle bacilli, with SITs commonly found in USA, Europe, South & Central America, and the Caribbean.

The five predominant spoligotypes in our study were: SIT33 (LAM3) 20.9% > SIT42 (LAM9) 10.2% > SIT67 (H3) 8.7% > SIT53 (T1) 7.8% > SIT376 (LAM3) 5.8%. A full description of the predominant spoligotypes found is shown in Table 1. Latin American-Mediterranean (LAM) strains constitute the most predominant lineage in our study, their total number being very high (113/206, 54.9%) with the following distribution: LAM1 n = 2, LAM2 n = 1, LAM3 n = 72, LAM4 n = 1, LAM6 n = 4, LAM9 n = 33. Other lineages found were Haarlem (H) (34/206, 16.5%) with the following distribution: H1 n = 8, H2 n = 3, H3 n = 23; the ill-defined T family (33/206, 16.0%): T with sub-lineage distinction n = 3, T1 n = 27, T4 n = 2, T5 n = 1; the X-clade (12/206, 5.8%): X1 n = 2, X3 n = 10; the S family n = 2; and the Beijing family n = 1. Furthermore, 11 strains had no known signatures.

**Table 1:** Description of predominant shared-types (SITs) in this study and their worldwide distribution according SITVIT2 database

SIT (Clade) **Octal Number**^**1**^	Total (%) in this study	**Distribution in Regions with ≥5% of a given SITs**^**2**^	**Distribution in countries with ≥5% of a given SITs**^**3**^
33 (LAM3) 776177607760771	43 (20.9)	AFRI-S 32.0, AMER-S 22.1, AMER-N 15.9, EURO-S 13.6, EURO-W 5.4	ZAF 32.0, USA 15.7, BRA 8.9, ESP 8.8, ARG 5.6, PER 5.5
42 (LAM9) 777777607760771	21 (10.2)	AMER-S 29.8, AMER-N 16.3, EURO-S 12.8, EURO-W 7.0, AFRI-N 5.1	USA 15.25, BRA 10.3, COL 7.9, ITA 6.7
53 (T1) 777777777760771	16 (7.8)	AMER-N 19.8, AMER-S 14.5, EURO-W 12.8, EURO-S 10.0, ASIA-W 8.7, AFRI-S 6.5	USA 17.3, ZAF 6.4, ITA 5.1
67 (H3) 777777037720771	18 (8.7)	AMER-N 46.3, AMER-C 35.2, AMER-S 13.0, CARI 5.6	USA 44.4, HND 33.3, GUF 12.9
92 (X3) 700076777760771	5 (2.4)	AFRI-S 50.3, AMER-N 23.0, AMER-S 9.0	ZAF 50.3, USA 20.6, BRA 5.4
206 (LAM9) 740777607760771	6 (2.9)	AMER-N 50.0, AMER-C 42.9, EURO-W 7.1	USA 50.0, HND 42.9, BEL 7.1
376 (LAM3) 376177607760771	12 (5.8)	AMER-N 44.7, AMER-C 25.5, AMER-S 21.3	USA 44.68, HND 25.53, VEN 17.0
546 (X3) 700036777560771	5 (2.4)	AMER-N 57.1, AMER-C 35.7, AMER-S 7.1	USA 57.1, HND 35.7, PER 7.1
1328 (H1) 777777034020771	5 (2.4)	AMER-C 55.6, CARI 22.2, AMER-N 22.2	HND 55.6, USA 22.2, HTI 22.2

^1 ^Predominant shared types (SITs) were defined as SITs representing 2% or more strains in this dataset (i.e., 4 strains or more strains in this study).

^2 ^Worldwide distribution is only reported for regions with ≥5% of a given SITs as compared to their total number in the SITVIT2 database. Regions description [16]: AFRI (Africa), AMER (Americas), ASIA (Asia), EURO (Europe), and CARIB (Caribbean), subdivided in: C (Central), N (Northern), S (Southern) and W (Western).

^3 ^Distribution by country is only shown for SITs with ≥5% in a given country: ARG (Argentina), BEL (Belgium), BRA (Brazil), COL (Colombia), ESP (Spain), GUF (French Guiana), HND (Honduras), HTI (Haiti), ITA (Italy), PER (Peru), USA (United States), VEN (Venezuela), ZAF (South Africa).

### RFLP results

**Figure 1:**
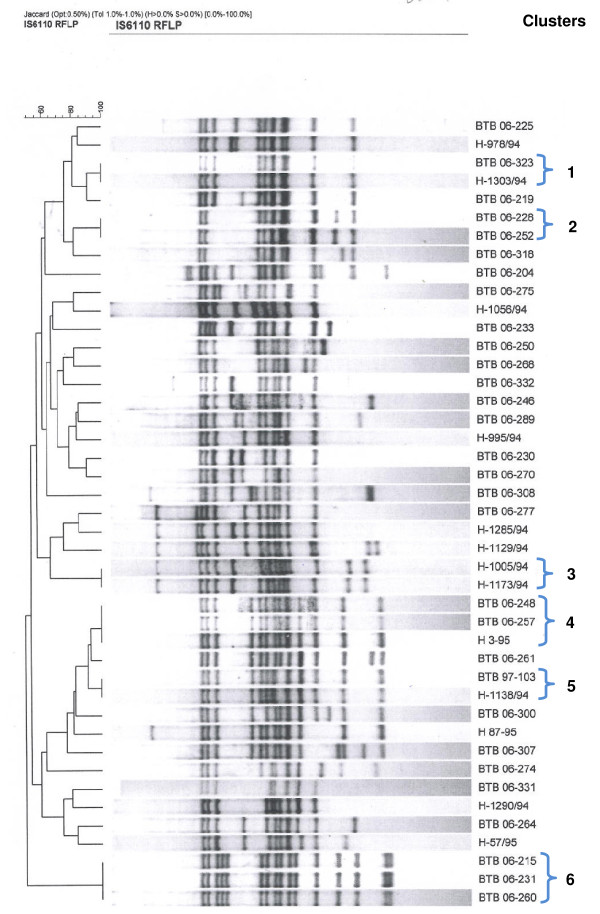
**Dendrogram of the 43 *M. tuberculosis *isolates belonging to SIT 33, LAM3**. The dendrogram displays the RFLP patterns and the isolate identification code of all the strains belonging to SIT 33. The clusters identified are designated with consecutive numbers.

All 43 strains within the main spoligotype cluster, belonging to the SIT 33 (LAM 3 genotype), were further characterized using RFLP IS*6110*. A total of 35 different fingerprints were identified, of which 29 (67.4%) were unique patterns. Six clusters with a total of 14 strains (2 to 3 strains per cluster) were identified (Figure 1). The average number of IS*6110 *copies was 12, with a range of 8-16 copies. Table 2 summarizes the clustered isolates' DST profile as well as their main demographic characteristics.

**Table 2:** Description of the RFLP clusters found among strains belonging to the SIT 33

RFLP cluster	Isolate	DST Profile				Sex	Age	Geographic Origin	
					
		STM	INH	RIF	EMB			Department	City
**1**	06-323	S	S	S	S	Male	34	Sta. Bárbara	Sta. Bárbara
	1303-94	S	S	S	S	Male	33	Choluteca	Marcovia
									
**2**	06-228	S	S	S	S	Male	29	Olancho	Juticalpa
	06-252	S	S	S	S	Female	62	Olancho	Catacamas
									
**3**	1005-94	R	R	R	R	Male	23	Fco. Morazán	Tegucigalpa
	1173-94	R	R	R	R	Male	29	Fco. Morazán	Tegucigalpa
									
**4**	06-248	S	S	S	S	Male	30	Cortés	San Pedro Sula
	06-257	S	S	S	S	Female	26	Fco. Morazán	Tegucigalpa
	3-95	S	S	S	S	Male	19	Fco. Morazán	Cedros
									
**5**	97-103	S	S	S	S	Male	20	Fco. Morazán	Tegucigalpa
	1138-94	S	S	S	S	Male	34	Fco. Morazán	Tegucigalpa
									
**6**	06-215	S	S	R	R	Male	57	Comayagua	Siguatepeque
	06-231	S	S	S	S	Male	22	Copán	La Entrada
	06-260	S	S	S	S	Female	22	Cortés	San Pedro Sula

### Population characteristics

Demographic information was available for 203 of the 206 TB cases (98.5%). Overall, 66.5% were male and 33.5% were female and the average age was 37 years (SD: 17 years) with an age range of 11 to 85 years. Half of the cases belonged to the 20-40 years age group. The patients represented all major geographical regions of the country. The HIV serological status was known for 36% of the cases; 14.7% were HIV-positive and 21.2% were HIV-negative. The majority of patients (95%) had smear-positive pulmonary TB. All 10 patients with extra-pulmonary TB were HIV-positive. A majority of the patients (56.2%) were new, previously untreated cases, 8.3% had been previously treated and in 35.5% of the cases, previous treatment status was unknown.

One hundred seventy-four isolates (85.7%) were pan-susceptible and 29 (14.3%) showed resistance to at least one of the first-line drugs. Multidrug resistance (MDR), defined as resistance to at least RIF and INH, was detected in 8 isolates. Of those, two were also resistant to EMB, one isolate was also resistant to STM and 2 were additionally resistant to both EMB and STM.

Nineteen strains were monoresistant (5 to INH, 2 to RIF, 12 to STM) and 2 isolates had other susceptibility patterns (one was RIF + STM resistant and the other was INH + STM resistant).

The single Beijing strain identified in this sample was susceptible to all drugs and was isolated from a female patient, 30 years of age, with pulmonary TB and unknown HIV status.

The distribution of spoligopatterns was not associated to gender or geographic origin (Table 3). When analyzing the mean age of patients harboring the predominant spoligotypes, we found that the mean age of cases belonging to SIT 33 was not significally different from the rest of the study population (37.8 vs. 36.9 years old, p = 0.79), nor was it different when compared to the mean age of cases belonging to the other predominant SITs (data not shown).

**Table 3:** Demographic characteristics of the study population and their association with spoligotype clustering

	Spoligotyping patterns			
			
Parameter	Clustered	Unique	OR (95%CI)	p-value
**Sex**				
Male	115	20	1.23	0.75
Female	56	12	(0.52 -2.88)	
**Age^1 ^**				
<35 years	96	18	0.94	0.97
>35 years	74	13	(0.40 - 2.17)	
**Tuberculosis localization**				
Pulmonary	164	29	2.42	0.20
Extra-pulmonary	7	3	(0.46 - 11.30)	
**HIV status**				
Positive	24	6		
Negative	36	7	NA^2^	0.76
Unknown	111	19		
**DST profile**				
Any Resistance	27	2	2.81	0.27
Susceptible	144	30	(0.60- 18.09)	

^1 ^Age information was missing for 2 out of 203 patients.

^2 ^NA = Not applicable

**Figure 2:**
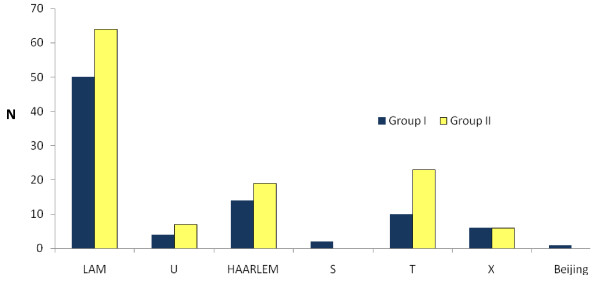
**Distribution of the spoligotype families**. N: total number of strains belonging to each spoligotype family. Group I: strains isolated between 1994 and 1998.Group II: strains isolated in 2002. LAM: Latin American Mediterranean. U: unknown.

The distribution of the spoligotype families between the two groups of isolates characterized was very similar to the overall distribution within the country, as shown in Figure 2. The overall proportion of clustered strains in this study was 84%, with a clustering rate of 80% in group I isolates and 87% in group II isolates.

## Discussion

This study included a total of 206 *M. tuberculosis *strains isolated from the same number of patients in Honduras and were collected during two different time periods (1994-1998 and 2002). All isolates were spoligotyped in order to identify the predominant genotypes within this subpopulation, as well as to compare the distribution of genotypes to the spoligotypes recorded in the SITVIT2 proprietary database of the Institut Pasteur de la Guadeloupe.

In Honduras, the LAM family was the most prevalent, with more than 50% of all patient isolates characterized belonging to this specific genotype. Thereafter the Haarlem and T clades were most common. The remaining genotypes contributed to only 13% of all isolates. These results are similar to previous studies in which these three genotypes have been seen to be predominant among TB cases in Mexico [22], South America [23-28] and the Caribbean [29]. However, there is limited information available regarding Central American MTC isolates, of which most information is based on TB cases detected among Central American immigrants in United States [30] and Canada [31]. Therefore, our study is providing a first characterization of the distribution of TB isolates within Honduras. Establishing such a baseline distribution of isolates will be useful for future genotyping investigations in Honduras as well as neighboring Central American countries.

According to the more recent genotype classification, which is based on large sequence polymorphisms in the MTC genome [30], the Euro-American lineage comprises the LAM, Haarlem, T and X spoligotyping-defined families. These phylogenetic clades are commonly included in the PGG 2 and 3 described by Sreevatsan *et al *[18,32]. Indeed, the most predominant clades in our study comprised PGG2/3 lineages: only 0, 5% of the isolates belonged to PGG1 (ancient lineages) as compared to 77% to the PGG2/3 (modern lineages).

These findings indicate that ongoing TB transmission in Honduras is mainly attributable to modern *M. tuberculosis *lineages. The evolutionary modern LAM-lineage was the most predominant among all lineages in this study and, having identified several LAM sub-lineages, was furthermore characterized by a high level of biodiversity. Indeed, of the 12 LAM- sub-lineages so far reported worldwide [14], a total of six (LAM1, 2, 3, 4, 6, and 9) were identified among this study's sample of Honduran TB patient isolates. A level of biodiversity was also observed within the PGG2/3 clades (X and H); however this was to a lesser extent. The "T" genotype has previously been defined to include strains that may not be classified in one of the established PGG2/3 genotypic lineages [14], was mostly represented in our study by its T1 sub-lineage.

All the spoligotypes not earlier described (orphans and new SITs) belong to PGG 2 and 3. The observation that a minimal number of PGG1 strains such as the EAI, CAS, Manu, Beijing (with only a single Beijing isolate, Table 2), *M. africanum *and *M. bovis *were identified in this study is noteworthy.

In Latin America, the prevalence of the Beijing genotype is low [22-25,33,34], especially if compared with Asian and East-European countries. The presence of only one, fully-susceptible, Beijing strain in our sample supports these findings.

To obtain a more complete and precise definition of isolate clusters, it is recommended to combine at least two genotyping techniques [35,36]. By using RFLP *IS*6110 to further characterize the major cluster identified in our study which comprised isolates from both group I and II, (the SIT 33 belonging to the LAM family), we observed a high degree of diversity among the 43 isolates analyzed. These findings were in agreement with the first genotyping study in Honduras [8]. Interestingly, the only RFLP cluster of MDR strains seen in this study belonged to group I, i.e., isolates from the mentioned first genotyping study [8]. This might indicate that the presence of MDR-TB in the country is due to acquired resistance.

A limitation within this study was the use of a relatively small sample size, representing approximately 1% of the total number of TB cases diagnosed in the country during the same period of time. Such sample size, can underestimate the clustering proportion [37,38]. Nevertheless, as explained below, we believe that the isolates characterized in this study were most likely representative of the overall distribution in the country. The isolates collected in 2002 (group II) were collected and cultured from smear positive Honduran patients using the cluster sampling method recommended by WHO/IUATLD guidelines for drug-resistance surveys [39]. Isolates in group I (samples from 1994-1998), although collected at the INCP in a specific setting, were geographically diverse as most chronic-diseased patients were referred to the national hospital from several regions in the country.

As information regarding epidemiological links between patients as well as risk behaviors could not be recovered, only a broad description of the genotypes' distribution within Honduras could be provided in this study. Another area of interest for further studies would be to assess the impact of HIV infection on TB transmission dynamics within Honduras.

## Conclusions

Spoligotyping has proven to be a useful genotyping method for the characterization of the MTC population structure in Honduras. The current study identified the LAM family as the most common spoligotype circulating in this setting. Furthermore, the high biodiversity, as demonstrated through the identification of several sub-lineages usgin RFLP, is a reflection of the LAM-family's adaptation to the host population over time.

However, prospective investigations, combined with contact-tracing and epidemiological linking, are required in order to obtain a more detailed molecular-epidemiological overview of TB transmission within Honduras.

## Competing interests

The authors declare that they have no competing interests.

## Authors' contributions

SR participated in the design of the study, performed and analyzed spoligotyping, collected epidemiologic data, conducted the statistical analysis and wrote the manuscript. LPG participated in the study design, carried out mycobacteriological diagnostics, isolation, identification and drug susceptibility testing of clinical isolates, collected epidemiological information, data analysis and provided critical comments for the manuscript. SG performed and analyzed RFLP; carried out bioinformatics analysis of spoligotyping and RFLP results. NR performed database analysis of the spoligotypes and helped draft the manuscript. SEH participated in the design of the study, analyzed the data and helped draft the manuscript. All authors read and approved the final version of the manuscript.

## Supplementary Material

Additional file 1 **Description of 16 orphan *M. tuberculosis *strains identified in Honduras**. Click here for file

Additional file 2 **Description of 44 shared spoligotypes (SITs) identified among *M. tuberculosis *isolates from Honduras**. This table summarizes genotypic clade designations and percentage distribution of all SITs present in this study. Click here for file
