# Supplementary material for: Molecular diversity of Mycobacterium tuberculosis isolates from patients with tuberculosis in Honduras
Source: BMC Microbiol. 2010 Aug 3;10:208. doi: 10.1186/1471-2180-10-208 (PMC2923133; doi:10.1186/1471-2180-10-208)
Supplement: Additional file 1 — Description of 16 orphan M. tuberculosis strains identified in Honduras. [file 1471-2180-10-208-S1.PDF]

<u>Additional file 1</u>. Description of 16 orphan *M. tuberculosis* strains identified in Honduras

Year	Strain Number	Spoligotype Description	Octal code	Clade[1]	Sex/ Age	Drug Resistance
2002	06-216	■■■■■■■■■■■■■■■■■■■□□□□□□□□■■■■■□□□□■■■□■■■	777777400760731	Unk	M/45	STM
2002	06-226	■■■■■■■■■■■■■■■■■■□□□□■■■■■■■■■□□□□□□□□■■■■	777777037740071	T1	M/44	-
1997	97-120	■■■■■■■■□□□■■■□■■■■■□□□□■■■■■■■■□□□□■■■□□■■	776167607760711	LAM3	M/36	-
1997	97-110	■■■□□□□□□□□□□■□■■■■■□□□□■■■■■■■■□□□□■■■□■■■	700027607760731	LAM3	M/61	-
2002	06-333	■■■■■■□□□■■■■■■■■■■■□□□□■■■■■■■■□□□□■■■■■■■	770777607760771	LAM9	M/50	-
2002	06-239	■■□□■■■■■■■■□■□■■■■■□□□□■■■■■■■■□□□□■■■■■■■	637727607760771	LAM2	F/27	RIF
1994	1240-94	□■■■■■■■□□□■■■■■■■■■□□□□□□□□■■■■□□□□■■■■■■■	376177600360771	LAM3	M/38	-
1994	1367-94	■■■■■■■■□□□□■■□■■□■■□■■□□■■□□□□■□□□□■■■■■■■	776066663020771	H3	M/33	-
2002	06-319	■■■■■■■■■■■■■■■■■■□□□□■■■■■■■■□■□□■■■■■■■■■	777777037723771	H3	M/49	-
2002	06-262	■■■■■■■■■■■■■■■■□□□□□□□□□□□□□□■■□□□□■■■■■■■	777774000060771	Unk	M/15	-
1994	1166-94	■■■■■■■■□□□■■■■■■■■■□□□□■■■□□□□□□□□□■■■■■■■	776177607000771	LAM3	M/33	-
2002	06-265	■■■■■■■■■■■■□■■■■■□■■■□□■■■■■■■■□□□□■■■■■■■	777737347760771	T	M/17	-
1997	97-104	■■■■□□□□■■■■■■□■□■□□□■□□■■■■□■■■□□□□■■■■■■■	741765047560771	Unk	M/37	-
2002	06-222	■■■■■□□□□□□□□□□□□□□□□□□□□□□□□□□□□□□□■■■■■■■	760000000000771	Unk	M/41	STM
2002	06-232	■■□□■■■■■■■■■■■■■■■■□□■■■■■■■■■■□□□□□□□□■■■	637777637760031	T1	F/29	-
1994	1064-94	■■■■□■■■■■■■■□□■■■■■■□□■■■■■■■■■■□□□□■■■■■■■	757717637760771	T1	F/30	-

[1] Clade designations according to SITVIT2 using revised SpolDB4 rules [14]; Unk= Unknown patterns. STM= streptomycin; RIF= rifampicin.
