# Supplementary material for: Molecular diversity of Mycobacterium tuberculosis isolates from patients with tuberculosis in Honduras
Source: BMC Microbiol. 2010 Aug 3;10:208. doi: 10.1186/1471-2180-10-208 (PMC2923133; doi:10.1186/1471-2180-10-208)
Supplement: Additional file 2 — Description of 44 shared spoligotypes (SITs) identified among M. tuberculosis isolates from Honduras. This table summarizes genotypic clade designations and percentage distribution of all SITs present in this study. [file 1471-2180-10-208-S2.PDF]

Additional File 2. Description of 44 shared spoligotypes (SITs) identified among *M. tuberculosis* isolates from Honduras.

SIT[1]	Spoligotype Description	Octal Number	Number (%) in this study	Clade[2]	Clustered vs. unique patterns[3]
1	□□□□□□□□□□□□□□□□□□□□□□□□□□□□□□□□□□■■■■■■■■■	000000000003771	1 (0.5)	Beijing	Unique
2	□□□□□□□□□□□□□□□□□□□□□□□□■□□□□□□■□□□□■■■■■■■	000000004020771	3 (1.5)	H2	Clustered
33	■■■■■■■■□□□■■■■■■■■■□□□□■■■■■■■■□□□□■■■■■■■	776177607760771	43 (20.9)	LAM3	Clustered
34	■■■■■■■■□□■■■■■■■■■■■■■■■■■■■■■■□□□□■■■■■■■	776377777760771	1 (0.5)	S	Unique
42	■■■■■■■■■■■■■■■■■■■■□□□□■■■■■■■■□□□□■■■■■■■	777777607760771	21 (10.2)	LAM9	Clustered
46	■■■■■■■■■■■■■■■■■■■■■■■■□□□□□□□□□□□□□□□□□□□	777777770000000	3 (1.4)	Unk	Clustered
47	■■■■■■■■■■■■■■■■■■■■■■■■■□□□□□□■□□□□■■■■■■■	777777774020771	1 (0.5)	H1	Unique
53	■■■■■■■■■■■■■■■■■■■■■■■■■■■■■■■■□□□□■■■■■■■	777777777760771	16 (7.8)	T1	Clustered
60	■■■■■■■■■■■■■■■■■■■■□□□□■■■■■■■■□□□□■■■□■■■	777777607760731	1 (0.5)	LAM4	Unique
64	■■■■■■■■■■■■■■■■■■■■□□□□■■■■□■■■□□□□■■■■■■■	777777607560771	1 (0.5)	LAM6	Unique
67	■■■■■■■■■■■■■■■■■■□□□□■■■■■■■■□■□□□□■■■■■■■	777777037720771	18 (8.7)	H3	Clustered
71	■■■■■■■■□□■■□■■■■■■■■■■■■■■■■■■■□□□□■■■■■■■	776337777760771	1 (0.5)	S	Unique
73	■■■■■■■■■■■■□■■■■■■■■■■■■■■■■■■■□□□□■■■□■■■	777737777760731	2 (0.9)	T	Clustered
92	■■■□□□□□□□□□■■■■■□■■■■■■■■■■■■■■□□□□■■■■■■■	700076777760771	5 (2.4)	X3	Clustered
111	■■■■■■■■□□□■■■□■■■■■□□□□■■■■■■■■□□□□■■■■■■■	776167607760771	2 (0.9)	LAM3	Clustered
118	■■■■■■■■■■■■■■□■■■■■■■■■■■■■■■■■□□□□■■■■■■■	777767777760771	2 (0.9)	T1	Clustered
119	■■■■■■■■■■■■■■■■■□■■■■■■■■■■■■■■□□□□■■■■■■■	777776777760771	1 (0.5)	X1	Unique
130	■■■■■■■■□□□■■■■■■■■■□□□□■■■■■■■■□□□□■■■□■■■	776177607760731	3 (1.5)	LAM3	Clustered
150	■■■■■■■■■■■■■■□■■■■■□□□□■■■■■■■■□□□□■■■■■■■	777767607760771	2 (0.9)	LAM9	Clustered
174	■■■■■■■■■■■■■■■■■■□□□□■■■■■■■■■■□□□□■■■■■■■	777777037760771	1 (0.5)	Unk	Unique
176	■■■■■■■■■■■■□■■■■■■■□□□□■■■■□■■■□□□□■■■■■■■	777737607560771	1 (0.5)	LAM6	Unique
206	■■■■□□□□□■■■■■■■■■■■□□□□■■■■■■■■□□□□■■■■■■■	740777607760771	6 (2.9)	LAM9	Clustered
218	■■■■■■■■■■■■□■■■■■■■■■■■■□□□□□□■□□□□■■■■■■■	777737774020771	1 (0.5)	H1	Unique
291	■■■■■■■■■■■■■■■■■■■■□■■■■■■■■■■■□□□□■■■■■■■	777777677760771	1 (0.5)	T1	Unique
294	■□■■■■■■■■■■■■■■■■■■■■■■■■■■■■□■□□□□■■■■■■■	577777777720771	3 (1.5)	H3	Clustered
376	□■■■■■■■□□□■■■■■■■■■□□□□■■■■■■■■□□□□■■■■■■■	376177607760771	12 (5.8)	LAM3	Clustered
546	■■■□□□□□□□□□□■■■■□■■■■■■■■■■□■■■□□□□■■■■■■■	700036777560771	5 (2.4)	X3	Clustered
635	□□□□□□□□□□□□□□□□□□□□□□□□■■■■□■■■□□□□■■■■■■■	000000007560771	1 (0.5)	LAM3	Unique
950	■■■□□□□□□□□□□■■■■■■■□□□□■■■■■■■■□□□□■■■■■■■	700037607760771	2 (0.9)	LAM3	Clustered
1156	■■□□■■■■□□■■■■■■■■■■□□□□■■■■■■■■□□□□■■■■■■■	636377607760771	2 (0.9)	LAM1	Clustered
1179	■■■■■■■■■■■■■■■■■□■■■■■■■■□□■■■■□□□□■■■■■■■	777776776360771	1 (0.5)	X1	Unique
1328	■■■■■■■■■■■■■■■■■■□□□□■■■□□□□□□■□□□□■■■■■■■	777777034020771	5 (2.4)	H1	Clustered
1354	■□■■■■■■□□□■■■■■■■■■□□□□■■■■■■■■□□□□■■■■■■■	576177607760771	1 (0.5)	LAM3	Unique
1536	■■■■■■■■□□■■■■■■■■■■□□□□■■■■■■■■□□□□■■■■■■■	776377607760771	1 (0.5)	LAM9	Unique
2151	■■■■■■■■■■■■■■■■■■□■■■□□■■■■■■■■□□□□■■■■■■■	777777347760771	2 (0.9)	T4	Clustered
3008	■■■■■■■■□■■■■■■■■■■■■■■■■□□□□□□■□□□□■■■■■■■	776777774020771	1 (0.5)	H1	Unique
3081*	■■■■■□■■□□□□■■■□■■■■■■■■■■■■■■■■□□□□■■■■■■■	766073777760771	3 (1.5)	T1	Clustered
3082*	■■■■■■■■□□□□■■■□□■■■■■■■■■■■■■■■□□□□■■■■■■■	776071777760771	2 (0.9)	T1	Clustered
3083*	□■■■■■■■□□□■■■■■■■■□□□□□■■■■■■■■□□□□■■■■■■■	376177407760771	2 (0.9)	LAM3	Clustered
3084*	□■■■■■■■□□□■■■■■■■■■□□□□■■■■■■■■□□□□□■■■■■■	376177607760371	2 (0.9)	LAM3	Clustered
3085*	■■■■■■■□■■■■■■■■■■■■□□□□■■■■■■■■□□□□■■■■■■■	775777607760771	2 (0.9)	LAM9	Clustered
3086*	■■■■□□□□■■■■■■■■□■□□□■□□■■■■□■■■□□□□■■■■■■■	741775047560771	3 (1.5)	Unk	Clustered
3087*	■■■■■■■■■■■■□□□□□■□□□□□□□■■■□■■■□□□□■■■■■■■	777701003560771	2 (0.9)	LAM6	Clustered
3088*	■□■■■■■■■■■■■■■■■■■■■■□■■■■■■■■■□□□□■■■■■■■	577777757760771	1 (0.5)	T5	Unique

[1] SIT followed by an asterisk indicates "newly identified shared-type". SITs 3081, 3082, 3084, 3086 and 3087 include strains with identical new patterns within this study. SITs 3083, 3085 and 3088 include Honduran new spoligotypes matching orphan patterns from Brazil, The Netherlands and Italy, respectively.

[2]Clade designations according to SITVIT2 using revised SpolDB4 rules [14]; Unk= Unknown patterns.

[3]Clustered patterns correspond to isolates with identical spoligotypes within this study; unique patterns did not match with another spoligotype identified in this study.
